# Supplementary material for: Increasing Temperature Activates TREK Potassium Currents in Vagal Afferent Neurons from the Nodose Ganglion
Source: Int J Mol Sci. 2025 Sep 18;26(18):9119. doi: 10.3390/ijms26189119 (PMC12470281; doi:10.3390/ijms26189119)
Supplement: Supplementary file 1 [file ijms-26-09119-s001.zip › ijms-3814599-supplementary.pdf]

Supplementary Materials:

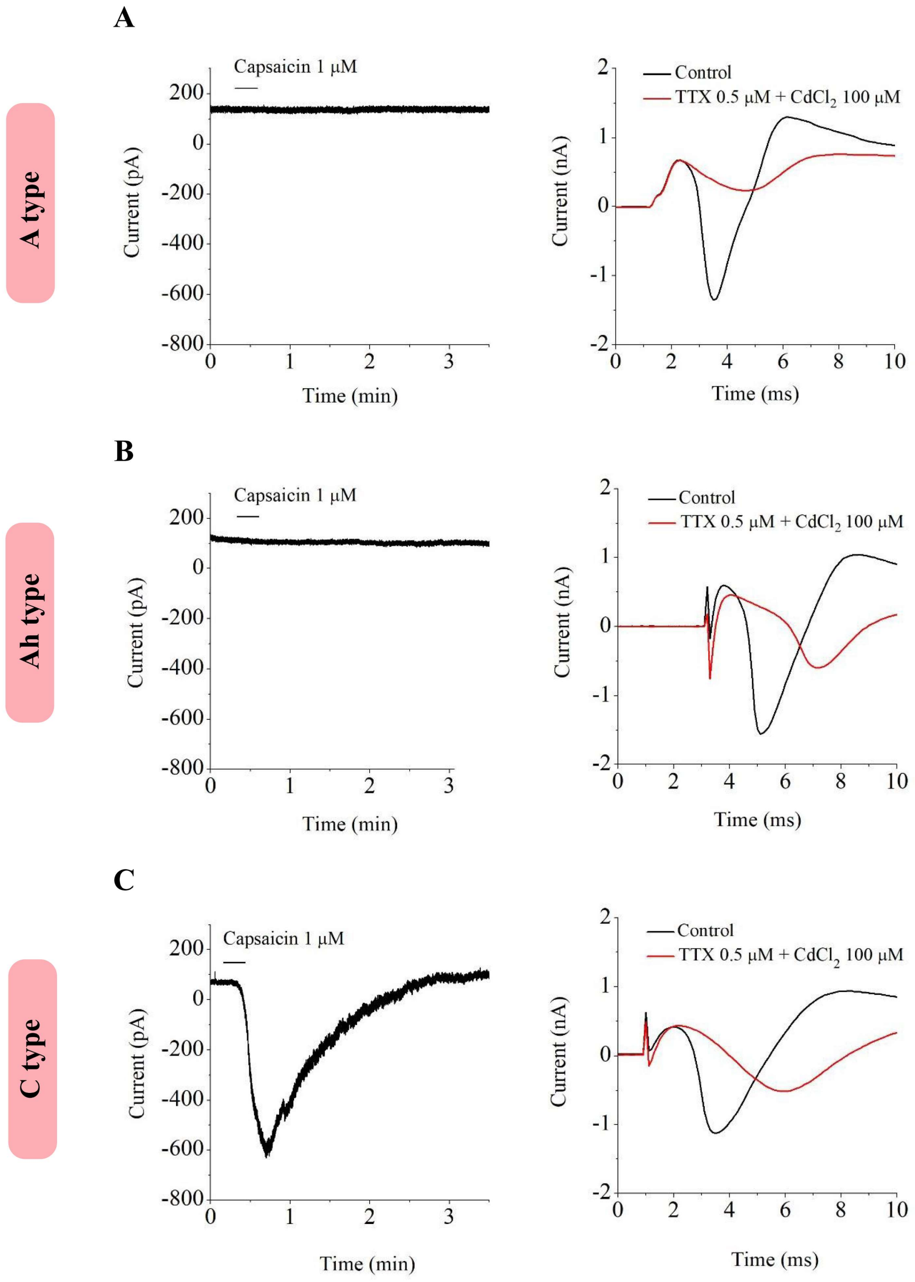

Figure S1. Pharmacological classification of neuronal types in NG from mice. A-type neurons ($n = 98$) showed as unresponsive to capsaicin and TTX-sensitive Na+ inward currents (**A**). Ah-type neurons ($n = 106$) did not respond to capsaicin and presented TTX-S and TTX-R Na+ inward currents (**B**). C-type neurons (92) showed an inward current in response to the application of capsaicin and presented TTX-S and TTX-R Na+ currents (**C**).

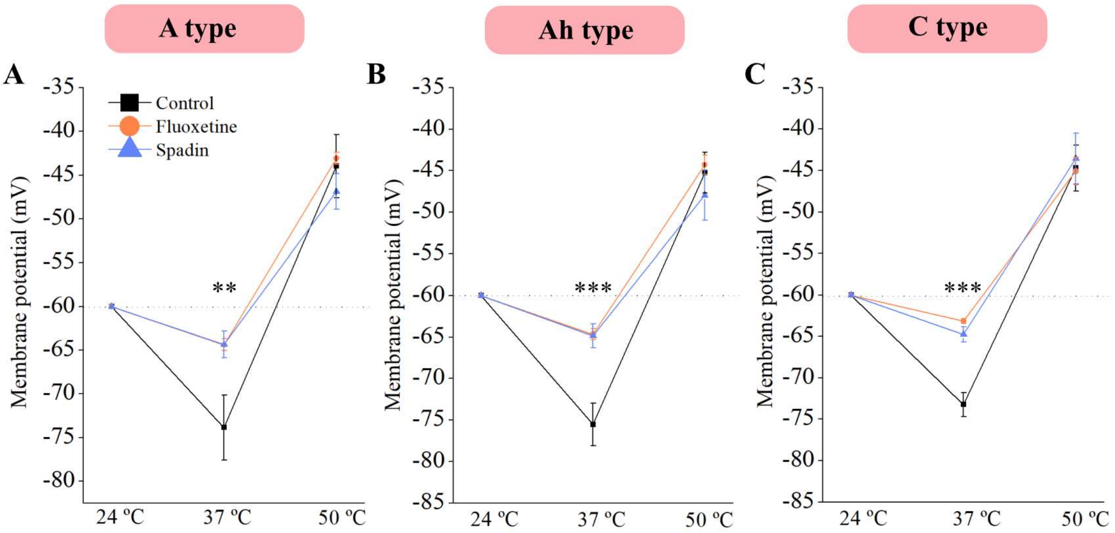

Figure S2: Heat-induced hyperpolarization at 37 °C and depolarization at 50 °C observed in the three NG neuronal subtypes. In current clamp gap-free protocol (neurons clamped at -60 mV), after the hyperpolarization produced observed at 37 °C (that is significantly blocked by fluoxetine 100 μM and spadin 1 μM), a depolarization is produced in A- (**A**), Ah- (**B**) and C-type neurons (**C**).
